# COMORBIDITY IMPACT ON SOCIAL FUNCTIONING AFTER HIP FRACTURE: THE ROLE OF REHABILITATION

**DOI:** 10.1590/1413-785220162404156874

**Published:** 2016

**Authors:** Natasa Radosavljevic, Dejan Nikolic, Milica Lazovic, Marija Hrkovic, Olivera Ilic-Stojanovic

**Affiliations:** 1. Institute for Rehabilitation, Belgrade, Serbia.; 2. University of Belgrade, Faculty of Medicine, Belgrade, Serbia.; 3. University Children's Hospital, Physical Medicine and Rehabilitation Department, Belgrade, Serbia.

**Keywords:** Hip fracture, Rehabilitation, Elderly, Follow-up studies

## Abstract

**Objective:**

To analyze the impact of rehabilitation treatment on social functioning in elderly patients after hip fracture during a rehabilitation program.

**Methods:**

This study included 203 patients with hip fracture. Four groups were analyzed on rehabilitation: Group 1, at admission, Group 2, at discharge, Group 3, three months after discharge and Group 4, six months after discharge. The analyzed parameters included: musculoskeletal, neurological and cognitive impairments. Impairment severity was graded by cumulative index rating scale for geriatrics (CIRS-G). Evaluation of social functioning was completed by social functioning component (SFC) from quality of life (SF-36) questionnaire.

**Results:**

There was a significant improvement in SF-36 SFC values for observed impairments from admission to six months after discharge for each severity degree (p<0.01), except for CIRS-G severity degree 4 for cognitive impairment, where significance was p<0.05. For the group of patients with musculoskeletal impairment, there was a significant difference between the values of SF-36 SFC concerning different severity degrees of CIRS-G only at six months after discharge (p<0.05). Patients with neurological or cognitive impairments have shown significant differences between the values of SF-36 SFC in regard to severity degrees of CIRS-G in all observational groups.

**Conclusion:**

Different degrees of observed impairments influence the degree of social functioning recovery in the elderly after hip fracture*.* ***Level of Evidence II, Prognostic Studies*** .

## INTRODUCTION

Functional decline, particularly in elderly population is associated closely with changes in expected individuals' quality of life. Previous studies have indicated potential predictors of functional decline in elderly.^1^ ^-^ ^3^ It was also suggested that there is a need for modifiable predictors evaluation, for the purpose of identification of elderly people who are at risk of functional and social decline.^4^

We hypothesized that increased severity of certain impairments among older individuals with hip fracture might have influence on social reintegration and social function decline in the period after the fracture. Further, we have postulated that implementation of optimal and multidisciplinary rehabilitation program might have a positive impact in preserving social functioning in the elderly after hip fracture with different comorbidities. Therefore, the aim of our study was to analyze the impact of rehabilitation treatment on social functioning in elderly patients after hip fracture during the rehabilitation program and in the period after discharge regarding their specific health impairments.

## MATERIAL AND METHODS

We performed longitudinal study that included 203 patients with hip fracture that were referred to Institute for Rehabilitation for post acute rehabilitation treatment after hip fracture. Prior to inclusion in the study, eligible participants were informed about the study protocol and patients' permissions were obtained. The study was approved by Institutional Review Board of Faculty of Medicine (440/IV-6) and followed the principles of good clinical practice.

Patients were grouped into four groups regarding the time of evaluation: Group 1- included patients at admission, Group 2- at discharge, Group 3- on 3 months post discharge and Group 4- on 6 months post discharge. Further impairments were analyzed: musculoskeletal, neurological and cognitive. For the gradation of impairment severity we used Cumulative index rating scale for geriatrics (CIRS-G) in the range between 0-4, where 0- refers to the condition with no impairment, 1- refers to mild, 2- moderate, 3- severe and 4- extremely severe impairment.^5^ ^,^ ^6^

For the evaluation of social functioning we used the social functioning component (SFC) from quality of life (SF-36) questionnaire.^7^

### Statistical interpretation

Patients distribution was presented as mean values (MV) with standard deviation (SD) for different degree of CIRS-G for every evaluated parameter (musculoskeletal, neurological and cognitive) in different times of observation. For the evaluation of statistical difference among these parameters we performed one way ANOVA and Mann-Whitney U test. Pearson's correlation coefficient was used to assess degree of correlation for every study parameter and same CIRS-G degree, between different times of observation. In order to evaluate and quantify variability that can be explained between different CIRS-G severity degrees and the values of social functioning of SF-36 questionnaire for analyzed CIRS-G parameters (musculoskeletal, neurological and cognitive) we introduced η2 = Sum of squares (Between groups) / Sum of squares (Total) x 100, where sum of squares were gained from the one-way ANOVA test and results were presented as percentage (%).^6^ Oneway ANOVA was used to evaluate statistical significance among different CIRS-G degrees at the same time of observation for every study parameter. Statistical significance was set at p<0.05.

## RESULTS

Evaluating defined severity degrees of CIRS-G in all groups of defined time periods (Groups 1-4) we found no significant differences in mean values of SF-36 SFC among musculoskeletal, neurological and cognitive impairments. (Table 1) Non significantly lowest values for SF-36 SFC is noticed for severity degree 4 for subjects with neurological impairment (SFC_Group1_=13.75±7.56) and cognitive impairment (SFC_Group1_=9.10±12.03) at admission. (Table 1) In this study there were no subjects with severity degree 4 of CIRS-G with musculoskeletal impairment. (Table 1) Non significantly highest values for SF-36 SFC is noticed for severity degree 0 for subjects with musculoskeletal impairment (SFC_Group4_=75.11±21.95), neurological impairment (SFC_Group 4_=72.80±23.96) and cognitive impairment (SFC_Group4_=77.47±19.86) 6 months post discharge. (Table 1).

**Table 1:** SF-36 SFC mean values in different time of observation for separate CIRS-G parameters regarding severity degree.

Evaluated groups	CIRS-G (degree)	Musculoskeletal impairment	Neurological impairment	Cognitive impairment	p value
Group 1 (MV ± SD)	0	28.07 ± 13.15	28.13 ± 14.66	30.16 ± 14.06	0.474^*^
	1	26.13 ± 16.39	27.21 ± 14.81	26.88 ± 14.86	0.952^*^
	2	24.93 ± 13.51	20.54 ± 13.52	21.31 ± 11.74	0.417^*^
	3	24.91 ± 14.18	20.59 ± 12.45	18.75 ± 10.72	0.249^*^
	4	-	13.75 ± 7.56	9.10 ± 12.03	0.267^**^
Group 2 (MV ± SD)	0	51.60 ± 13.98	50.29 ± 15.90	53.06 ± 14.33	0.345^*^
	1	48.67 ± 18.40	50.00 ± 17.68	48.75 ± 17.40	0.962^*^
	2	46.28 ± 14.98	46.43 ± 15.06	43.75 ± 14.68	0.774^*^
	3	44.40 ± 14.79	42.29 ± 12.78	37.50 ± 12.86	0.253^*^
	4	-	29.69 ± 11.45	30.30 ± 14.59	0.968^**^
Group 3 (MV ± SD)	0	64.96 ± 18.16	63.28 ± 20.16	67.34 ± 17.04	0.232^*^
	1	58.50 ± 23.63	61.77 ± 21.41	60.31 ± 20.19	0.828
	2	59.60 ± 19.58	51.79 ± 23.44	54.21 ± 22.80	0.422^*^
	3	56.71 ± 21.56	52.21 ± 20.84	45.83 ± 18.69	0.221^*^
	4	-	37.50 ± 16.37	27.80 ± 19.35	0.230^**^
Group 4 (MV ± SD)	0	75.11 ± 21.95	72.80 ± 23.96	77.47 ± 19.86	0.252^*^
	1	64.63 ± 27.71	63.24 ± 27.41	69.06 ± 22.64	0.630^*^
	2	69.79 ± 25.13	56.21 ± 28.02	57.69 ± 31.04	0.143^*^
	3	59.57 ± 26.96	57.35 ± 25.79	45.14 ± 21.07	0.153^*^
	4	-	34.38 ± 17.36	29.45 ± 22.07	0.478^**^

*Oneway ANOVA; **Mann-Whitney U test.

There is a significant improvement shown to be in SF-36 SFC values for observed impairments throughout follow-up, from admission to 6 months post discharge for every severity degree at the level of p<0.01, except for CIRS-G severity degree 4 for cognitive impairment, where significance was at level p<0.05. (Table 2).

**Table 2:** Statistical interpretation of SF-36 SFC during the rehabilitation treatment for same CIRS-G severity degree.

Evaluated groups	CIRS-G (degree)	Musculoskeletal impairment		Neurological impairment		Cognitive impairment	
		N	(F value) ^†^	N	(F value) ^†^	N	(F value) ^†^
Groups 1-4	0	62	87.168**	147	151.851**	109	168.584**
	1	75	44.313**	17	10.828**	40	36.919**
	2	37	39.071**	14	8.196**	26	15.219**
	3	29	17.889**	17	12.685**	18	10.672**
4	0	-	8	4.704**	10	3.344*

† Oneway ANOVA; *p<0.05; **p<0.01.

In Table 3, there is a significant correlation of SF-36 SFC shown to before musculoskeletal, neurological and cognitive impairments for same severity degree of CIRS-G in different times of observations. The highest correlation coefficient for the group with musculoskeletal impairment is for CIRS-G severity degree 1, between Group 2 and Group 3 (R=0.919), for subjects with neurological impairment for CIRS-G severity degree 2, between Group 1 and Group 2 (R=0.919) and for subjects with cognitive impairment for CIRS-G severity degree 2, between Group 2 and Group 3 (R=0.936). (Table 3)

**Table 3:** Correlations of SF-36 SFC values in defined severity degree of CIRS-G regarding the time of observation.

CIRS-G (degree)	Evaluated groups	Musculoskeletal impairment^†^	Neurological impairment^†^	Cognitive impairment^†^
0	Groups 1/2	0.783	0.820	0.794
	Groups 1/3	0.622	0.742	0.696
	Groups 1/4	0.581	0.696	0.633
	Groups 2/3	0.765	0.895	0.862
	Groups 2/4	0.765	0.843	0.789
1	Groups 1/2	0.867	0.858	0.846
	Groups 1/3	0.837	0.837	0.735
	Groups 1/4	0.789	0.765	0.653
	Groups 2/3	0.919	0.903	0.904
	Groups 2/4	0.877	0.766	0.814
2	Groups 1/2	0.796	0.919	0.786
	Groups 1/3	0.740	0.785	0.801
	Groups 1/4	0.655	0.683	0.791
	Groups 2/3	0.903	0.905	0.936
	Groups 2/4	0.828	0.826	0.864
3	Groups 1/2	0.876	0.816	0.800
	Groups 1/3	0.849	0.792	0.826
	Groups 1/4	0.849	0.715	0.712
	Groups 2/3	0.892	0.820	0.765
	Groups 2/4	0.825	0.776	0.746
4	Groups 1/2	-	0.851	0.912
	Groups 1/3	-	0.830	0.946
	Groups 1/4	-	0.646	0.924
	Groups 2/3	-	0.715	0.868
	Groups 2/4	-	0.646	0.843

† Pearson correlation coefficient.

For the group of patients with musculoskeletal impairment there is a significant difference between the values of SF-36 SFC concerning different severity degrees of CIRS-G only 6 months post discharge (Group 4; p<0.05). (Table 4) While for the subjects with neurological or cognitive impairments, there is a significant difference between the values of SF-36 SFC in regard to severity degrees of CIRS-G in all observational groups (Groups 1-4). (Table 4) The effects size of the CIRS-G severity degree on SF-36 SFC values is weaker for all comorbidity parameters in the group at admission (musculoskeletal impairement-η^2^_Group1_=0.75; neurological impairement-η^2^_Group1_=6.49 and cognitive impairement-η^2^_Group1_=14.60). (Table 4) The highest effects of CIRS-G severity degree on SF-36 SFC values are noticed for all comorbidity parameters in the group 6 months post discharge (musculoskeletal impairement-η^2^_Group4_=4.55; neurological impairement-η^2^_Group4_=12.32 and cognitive impairement-η^2^_Group4_=27.20). (Table 4) 

**Table 4:** Statistical interpretation of SF-36 SFC values changes in different time of observation for separate CIRS-G parameters regarding severity degree.

SF-36 SFC	Musculoskeletal impairment			Neurological impairment		Cognitive impairment	
	F value ^†^	η^2^ (%)	F value ^†^	η^2^ (%)	F value ^†^	η^2^ (%)
Group 1	0.498	0.75	3.434*	6.49	8.465**	14.60
Group 2	1.642	2.42	4.150**	7.74	9.374**	15.92
Group 3	1.487	2.19	4.568**	8.45	14.718**	22.92
Group 4	3.165*	4.55	6.956**	12.32	18.498**	27.20

† Oneway ANOVA; *p<0.05; **p<0.01.

## DISCUSSION

It is pointed out from the results of our study that the social functioning values measured by the SF-36 in elderly after hip fracture are not significantly influenced by the type of observed impairment (musculoskeletal, neurological and cognitive), while severity of the degree of CIRS-G influences significantly on social functioning recovery in groups of patients with neurological and cognitive impairments throughout entire follow-up and on long-term recovery for the group of patients with musculoskeletal impairment. These observations are consistent with previous studies which indicated comorbidity as the important predictor of mobility in patients with hip fracture.^8^ Previously it was stressed out as well, that cognitive function is among most important prognostic factors in rehabilitation outcome for these patients.^9^

Numerous studies have highlighted the positive effects of rehabilitation treatment in functional outcome of elderly patients after hip fracture.^10^ ^-^ ^12^ This is of particular importance since most of these patients suffer from, to the certain extent, a decrease in physical functioning and thus overall quality of life that might influence social functioning as well. Therefore, improvement in various functional abilities that would alter mobility could possibly influence improvement as well, in social functioning of elderly after hip fracture. The benefit of extended exercise rehabilitation for these patients was stressed out in the study of Auais et al.,^13^ where authors noticed that such program has an impact on different functional abilities. Our results are consistent with previous observation, and have proved the efficacy of rehabilitation program in social function improvement, independent of presence of observed joined impairments. 

We have demonstrated that the increase in CIRS-G severity degree for the group of patients with musculoskeletal impairment correlates closely with social functioning only for the group of patients 6 months post discharge, while for the group of patients with neurological and cognitive impairments CIRS-G severity degree correlated closely with social functioning through entire follow-up of such patients. The effects size of CIRS-G severity degrees for cognitive impairment on SFC SF-36 component values were among highest, particularly in the groups 3 and 6 months post discharge (η^2^_Group 2_=22.92% and η^2^_Group 3_=27.20%). The weakest effects size of CIRS-G severity degrees for musculoskeletal impairment on SFC SF-36 component values were through entire follow-up.

Our findings are consistent with previous reports, particularly concerning cognitive impairment, since we have noticed that it is negative predictor of improvement in social functioning and thus recovery during rehabilitation program (short term) and over the period of follow-up (long term).^14^ ^,^ ^15^

## CONCLUSIONS

Different degrees of observed impairments (comorbidities) influence the degree of social functioning recovery in elderly after hip fracture. Therefore, individual approach as well as continuous implementation of rehabilitation program is of great importance in recovery period after hip fracture. Both short term and long term rehabilitation programs are shown to be beneficial, thus they should be mandatory for treatment of these patients particularly in domains of social functioning improvement regardless of present comorbidity, ultimately affecting and improving the one's quality of life.
